# Dopaminergic Modulation of Forced Running Performance in Adolescent Rats: Role of Striatal D1 and Extra-striatal D2 Dopamine Receptors

**DOI:** 10.1007/s12035-020-02252-2

**Published:** 2021-01-04

**Authors:** Angel Toval, Daniel Garrigos, Yevheniy Kutsenko, Miroljub Popović, Bruno Ribeiro Do-Couto, Nicanor Morales-Delgado, Kuei Y. Tseng, José Luis Ferran

**Affiliations:** 1 Department of Human Anatomy and Psychobiology, Faculty of Medicine, University of Murcia, Murcia, Spain; 2 Institute of Biomedical Research of Murcia – IMIB, Virgen de la Arrixaca University Hospital, University of Murcia, Murcia, Spain; 3 Department of Human Anatomy and Psychobiology, Faculty of Psychology, University of Murcia, Murcia, Spain; 4 Department of Histology and Anatomy, Faculty of Medicine, University Miguel Hernández, Sant Joan d’Alacant, Spain; 5 Department of Anatomy and Cell Biology, University of Illinois at Chicago, Chicago, IL USA

**Keywords:** Habituation, Endurance, Dopaminergic system, Exercise capacity, Motor learning, Central fatigue

## Abstract

Improving exercise capacity during adolescence impacts positively on cognitive and motor functions. However, the neural mechanisms contributing to enhance physical performance during this sensitive period remain poorly understood. Such knowledge could help to optimize exercise programs and promote a healthy physical and cognitive development in youth athletes. The central dopamine system is of great interest because of its role in regulating motor behavior through the activation of D1 and D2 receptors. Thus, the aim of the present study is to determine whether D1 or D2 receptor signaling contributes to modulate the exercise capacity during adolescence and if this modulation takes place through the striatum. To test this, we used a rodent model of forced running wheel that we implemented recently to assess the exercise capacity. Briefly, rats were exposed to an 8-day period of habituation in the running wheel before assessing their locomotor performance in response to an incremental exercise test, in which the speed was gradually increased until exhaustion. We found that systemic administration of D1-like (SCH23390) and/or D2-like (raclopride) receptor antagonists prior to the incremental test reduced the duration of forced running in a dose-dependent manner. Similarly, locomotor activity in the open field was decreased by the dopamine antagonists. Interestingly, this was not the case following intrastriatal infusion of an effective dose of SCH23390, which decreased motor performance during the incremental test without disrupting the behavioral response in the open field. Surprisingly, intrastriatal delivery of raclopride failed to impact the duration of forced running. Altogether, these results indicate that the level of locomotor response to incremental loads of forced running in adolescent rats is dopamine dependent and mechanistically linked to the activation of striatal D1 and extra-striatal D2 receptors.

## Introduction

A healthier life expectancy is associated with physical activity [1, 2], yet the biological mechanisms underlying such an effect remain poorly understood. Physical activity is a powerful enhancer of brain plasticity and positively impacts a broad set of cognitive and motor functions [3–6]. In fact, the maximal amount of physical activity an individual can perform (defined as exercise capacity) [7] is a good predictor of health status and physical fitness, and it is commonly used for individualized exercise programs [8–10]. Of particular interest is the adolescent period, which is characterized by key behavioral and biological changes in physical development and brain circuit maturation [11–17]. In order to enhance the potential and development of adolescent athletes, the different stages of exercise programs (e.g., physical literacy, motor coordination skills) need to be adapted to the biological maturity of this sensitive period [11, 12]. Thus, understanding the underlying neural processes that contribute to the enhancement of physical performance during adolescence will allow to act upon such precise neurobiological mechanism to optimize training programs in youth athletes.

Among the different neural processes contributing to brain maturation during adolescence is the central dopamine system [13, 14, 18], which also plays a crucial role in modulating a wide variety of cognitive and motor behavior including locomotor activity, learning, and memory [19–23]. At the cellular level, dopamine exerts its excitatory and inhibitory actions on targeted neurons depending on which receptor is being activated. There are five different types of dopamine receptors, which can be grouped into two major families: D1-like (constituted by D1R and D5R) and D2-like (constituted by D2R, D3R, and D4R). D1-like receptor family has an excitatory effect, whereas D2-like receptors have an inhibitory effect [24–26]. Among the main targets of the dopamine system are the corticostriatal circuits, which are composed of striatal output neurons containing D1 (direct pathway) and D2 (indirect pathway) receptors. Direct and indirect pathways are part of the cortico-basal ganglia-thalamo-cortical loop. Direct pathway projects from striatum to substantia nigra pars reticulata (SNr) and the internal portion of globus pallidus and facilitates initiation and execution of movement. Indirect pathway sends their primary striatal projections to the external portion of globus pallidus (GP) leading to inhibition of movement [20, 22, 23]. Pharmacological manipulation of dopamine receptors has shown effects on spontaneous motor activity and voluntary running [20, 27–34]. Moreover, increments in dopamine content and release have been observed in response to physical activity [3, 35–38], and genetic and pharmacological studies have suggested that a reduction in dopamine function could limit the activation of motor circuits and negatively impact the exercise capacity [3, 39–41].

The aim of the present study is to determine whether the dopamine system modulates exercise capacity during adolescence using a rodent model of forced running wheel we recently implemented. Briefly, rats were exposed to an 8-day period of habituation in the running wheel before assessing their locomotor performance in response to an incremental load of forced running [42, 43]. Both systemic and striatal administration of dopamine D1 (SCH23390) and D2 (raclopride) receptor antagonists were delivered 15–30 min prior to the onset of the incremental test, and changes in the level of locomotor performance were compared.

## Methods

All the experimental procedures were approved by the Ethical Committee for Animal Research (CEEA) of the University of Murcia according to the Spanish regulation on the use of animals for scientific purposes (Royal Degree 53/2013, Law 32/2007) and European Union directives (86/609/EEC).

### Animals

Adolescent male Sprague-Dawley rats (Laboratory Animal Facilities at the University of Murcia) aged P32–50 days were group housed (3 rats per cage) and randomly assigned to the different experimental groups. The randomization schedule was generated by using the website www.randomizer.org. Temperature and relative humidity were kept at 21–23 °C and 55 ± 5%. Chow food and water were provided ad libitum. The light cycle was kept in a 12:12-h light/dark (dark period from 8AM to 8PM), and all the procedures were performed during the dark phase. All the rats were handled daily for 1 min during 5 days before the beginning of the habituation protocol to get familiarized with the researcher and the experimental conditions.

Rats subjected to stereotaxic surgery (at P41/42) were individually housed after surgery and during the exercise habituation protocol.

### Exercise Habituation and Incremental Test

An 8-day exercise habituation protocol was implemented using a forced running wheel system (Lafayette-Campden, model 8085A). Speed and time of running were progressively increased throughout the sessions of the protocol, from 5 at 0 m/min the first day to 30 min running at 9 m/min the last day, as described in Toval et al. (2017, 2020) [42, 43]. Non-habituated rats remained in locked wheels, without any exercise stimulus, for the same time as the habituated groups.

**Fig. 1:**
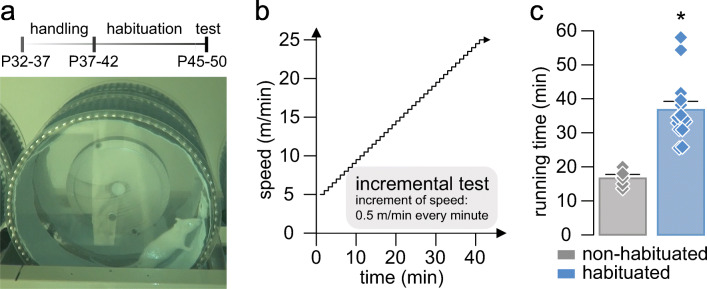
Effects of habituation on the locomotor response during an incremental exercise test. (**a**) Timeline of the experiments and picture of an adolescent rat running in the forced wheel during the dark period. (**b**) Speed variation (*y*-axis) along time (*x*-axis) during the incremental exercise test. (**c**) Average of the total running time spent during the incremental exercise test comparing non-habituated (gray bar) and habituated (blue bar) rats injected with vehicle (NaCl, 0.9%, i.p.). Data: non-habituated: 16.65 ± 1.11 min (*n* = 5), and habituated: 36.75 ± 2.52 min (*n* = 14). *t*_17_ = 4.6, *p < 0.001, unpaired *t* test. Diamonds represent individual values

Twenty-four hours after the last session of the habituation protocol, motor performance was evaluated by an incremental exercise test in all the animals. During the test, running speed was gradually increased 0.5 m/min every minute from a starting speed of 5 m/min (Fig. 1b, modified from Toval et al. (2017, 2020) [42, 43]). The test was concluded when the rat was unable to maintain a regular running path (e.g., crawling, jumping, or rolling inside the wheel) for 20 s in a row. The first 5 min were considered as warm-up phase, since some rats show transient irregular running paths at the beginning of the test. Thus, the mentioned criteria to stop the test were applied after these first 5 min. The decision of concluding the test for every rat was achieved through consensus of two experimented researchers.

### Drug Administration

To evaluate whether the dopaminergic system plays a role in mediating the motor performance, dopamine receptors were pharmacologically blocked during the incremental test by systemic and intrastriatal administration of the dopamine D1 receptor antagonist SCH23390 (Hello Bio, HB1643) and the D2 receptor antagonist raclopride (Sigma, 98185-20-7). For systemic administration, drugs were diluted in NaCl, 0.9%, and injected intraperitoneally (i.p.) in a volume of 0.5 ml. All systemic injections were applied 20 min before the incremental test. For intrastriatal administration, drugs were diluted in artificial cerebrospinal fluid (aCSF) composed of (in mM) 122.5 NaCl, 3.5 KCl, 25 NaHCO_3_, 1 NaH_2_PO_4_, 2.5 CaCl_2_, 1 MgCl_2_, 20 glucose, and 1 ascorbic acid (pH: 7.4, 295–305 mOsm). All micro-infusions were performed bilaterally with a volume of 1 μL per hemisphere at a rate of 0.5 μL/min using infusion cannulas (Plastics One, C315IA/SPC). After 2 min of infusion, an additional 1.5 min were given until the infusion cannula is removed, for a complete spreading of the drug. All intrastriatal infusions were applied 15–30 min before the incremental test.

### Experimental Design

First, to assess the effects of habituation on locomotor performance, non-habituated and habituated rats were subjected to the incremental exercise test after systemic administration of vehicle (NaCl 0.9%, i.p.).

**Fig. 2:**
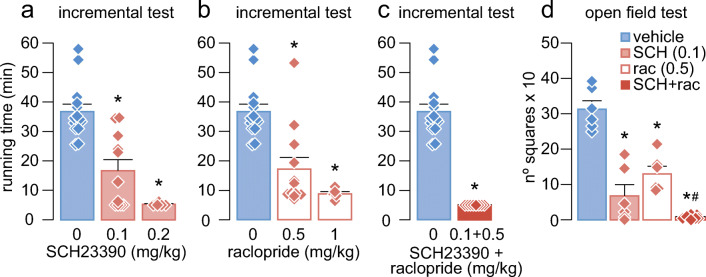
Systemic blockade of D1 and D2 receptors during the incremental exercise test and open field. (**a**) Mean running time during the incremental test after systemic (i.p.) injection of the D1 antagonist SCH23390 (0.1 and 0.2 mg/kg). Data: SCH23390: 0 mg/kg: 36.75 ± 2.52 min (*n* = 14, blue bar), 0.1 mg/kg: 16.67 ± 3.74 min (*n* = 11, red bar), and 0.2 mg/kg: 5.23 ± 0.23 min, (*n* = 5, red bar). *p < 0.0001 vs 0 mg/kg, Tukey post hoc test after significant one-way ANOVA, *F*_2,27_ = 23.2, *p* < 0.0001. (**b**) Mean running time during the incremental test after systemic (i.p.) injection of the D2 antagonist raclopride (0.5 and 1 mg/kg). Data: 0 mg/kg: 36.75 ± 2.52 min (*n* = 14, blue bar), 0.5 mg/kg: 17.19 ± 3.99 min (*n* = 12, white bar), and 1 mg/kg: 8.78 ± 0.78 min (*n* = 5, white bar). *p < 0.001 vs 0 mg/kg, Tukey post hoc test after significant one-way ANOVA, *F*_2,28_ = 17, *p* < 0.0001. (**c**) Mean running time during the incremental test after systemic (i.p.) injection of SCH23390 (0.1 mg/kg) plus raclopride (0.5 mg/kg). Data: 0 mg/kg: 36.75 ± 2.52 min (*n* = 14, blue bar) and SCH23390 + raclopride: 5 ± 0 min (*n* = 8, red bar). *t*_20_ = 9.4, *p < 0.0001, unpaired *t* test. (**d**) Locomotor activity during an open field test, comparing the effects of systemic (i.p.) injections of vehicle (blue bar), SCH23390 (0.1 mg/kg, light red bar), raclopride (0.5 mg/kg, white bar), and SCH23390 (0.1 mg/kg) plus raclopride (0.5 mg/kg, dark red bar). Data: vehicle: 313 ± 23.86 (*n* = 6), SCH23390: 67.67 ± 31.85 (*n* = 6), and raclopride: 130 ± 21.25 (*n* = 6), SCH23390 + raclopride: 7.14 ± 1.95 (*n* = 7). *p < 0.0001 vs vehicle, #*p* = 0.0027 vs raclopride, Tukey post hoc test after significant one-way ANOVA, *F*_3,21_ = 37.6, *p* < 0.0001. Diamonds represent individual values

Next, we aimed to find out the role of dopamine receptors in mediating the locomotor performance. For that purpose, D1-like receptors were selectively blocked by injecting SCH23390 (0.1 and 0.2 mg/kg, i.p.) (Fig. 2a), and D2-like receptors were selectively blocked by injecting raclopride (0.5 and 1 mg/kg, i.p.) (Fig. 2b) before the incremental test. These doses have been previously shown to induce effects on motor behaviors [36, 44]. After that, we injected a combination of SCH23390 (0.1 mg/kg, i.p.) plus raclopride (0.5 mg/kg, i.p.) given together using the lowest effective dose when given alone (Fig. 2c). The running time spent during the test was compared with habituated rats injected with vehicle (NaCl 0.9%, i.p.) (Fig. 2).

**Fig. 3:**
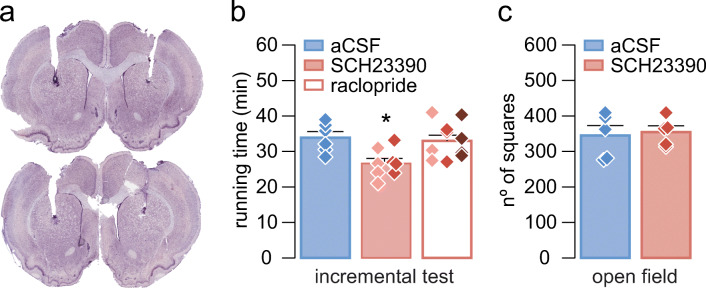
Intrastriatal blockade of D1 and D2 receptors during the incremental exercise test and open field. (**a**) Examples of Nissl-stained coronal sections showing the site of injection. The image above shows an example of a rat injected with raclopride and the image below a rat injected with SCH23390. (**b**) Mean running time during the incremental exercise test after intrastriatal administration of aCSF (blue bar), SCH23390 (red bar, 10 μM,  and 100 μM, ), or raclopride (white bar, 20 μM,  200 μM,  and 5 mM, ). Data from the different concentrations were pooled for treatment comparison. Data: aCSF: 33.87 ± 1.7 min (*n* = 6), SCH23390, 10 and 100 μM: 26.64 ± 1.39 min (*n* = 8), and raclopride, 20 μM, 200 μM, and 5 mM (*n* = 10): 33.01 ± 1.62. *p < 0.02 vs aCSF, Tukey post-hoc test after significant one-way ANOVA, *F*_2,21_ = 5.9, *p* < 0.01. Diamonds represent individual values. (**c**) Locomotor activity during an open field test, comparing the effects of intrastriatal administration (i.s.) of aCSF and SCH23390 (100 μM). Data: aCSF (i.s.): 344.8 ± 28.14 (*n* = 5, blue bar) and SCH23390 (i.s.): 354.6 ± 17.51 (*n* = 5, red bar). *t*_8_ = 0.3, *p* = 0.775, unpaired *t* test

Finally, in order to determine whether the observed role of the dopaminergic system in motor performance was mediated by striatal neurons, intracerebral cannulas were implanted in the dorsal striatum (at P41/P42) with the aim to specifically block striatal dopamine receptors. One week after the implantation of the cannula, rats were exposed to the habituation protocol, and, prior the incremental test, striatal D1 and D2 dopamine receptors were blocked by local administration of SCH23390 (D1 antagonist, 10 μM and 100 μM) or raclopride (D2 antagonist, at 20 μM, 200 μM, and 5 mM) (Fig. 3a, b). These doses have been previously shown to induce changes in the local field potential (LFP) activity of the cortex [45].

Furthermore, we assessed whether the observed effect of the dopamine system during forced running was also affecting ambulatory locomotor activity, evaluated by an open field test, as described below (Fig. 2d, 3c).

### Intrastriatal Implantation of Guide Cannulas

Stereotaxic surgery was conducted under inhalation of isoflurane anesthesia (5% for induction and 2–3% for maintenance with O_2_ flow at 0.8 L/min). Rats were injected with a non-steroidal anti-inflammatory drug (meloxicam, s.c. 1.5 mg/kg), with an antibiotic (enrofloxacin, i.m. 10 mg/kg) and with a local anesthetic (bupivacaine mixed with epinephrine, s.c. local, 1 mg/kg; epinephrine 1:100000). Twenty-four hours after the surgery, the rats were given another injection of meloxicam (s.c. 1.5 mg/kg). These treatments were meant to achieve analgesia and improve animal welfare after surgery. Guide cannulas (Plastics One, C315GA/SPC, 8 mm) were implanted bilaterally into the dorsal striatum at the following coordinates: anteroposterior (AP), − 0.7 mm; lateral (L), + 3.0 mm; and dorso-ventral (V), − 3.6 mm (Paxinos and Watson, 2007 [46]) and protected with dummy cannulas (Plastics One, C315DC/SPC, 8 mm, without projection). After 1 week of post-operative recovery, with daily handling, rats started the habituation protocol at P49.

The day before the incremental test, the dummy cannula was replaced with a longer one (Plastics One, C315IA/SPC9) that protruded 0.5 mm beyond the tip of the guide cannula to reduce possible damage caused by the infusion cannula and to facilitate drug spreading. After the experiment, rats were sacrificed, and cannula placement was confirmed by Nissl-stained coronal sections (Fig. 3a). Rats with off-target cannulations were excluded from the study.

### Open Field Test

The open field test was performed in a white plywood box (100 × 100 × 40 cm) under 4 lx intensity and recorded using a video camera. The floor was divided into 25 squares (20 × 20 cm). All rats were started the test at the same corner of the box 20 min after systemic (i.p.) administration of SCH23390 (0.1 mg/kg), raclopride (0.5 mg/kg), a combination of SCH23390 (0.1 mg/kg) plus raclopride (0.5 mg/kg,) or vehicle (NaCl 0.9%) (Fig. 2d) and 15 min after intrastriatal infusion of SCH23390 (100 µM, diluted in aCSF) and vehicle (aCSF) (Fig. 3c). For the open field test, the lowest effective dose during the incremental test in the forced wheel was used. The behavior of the animals was monitored for 15 min. The rats were then removed from the open field and returned to their cages. The surface was cleaned with 70% ethanol before each test. Ambulatory locomotor activity was measured by the total number of squares entered by the rats (Figs. 2d and 3c) [47].

### Statistical Analysis

Statistical analysis was performed using SPSS v25. All data are presented as mean ± standard error of the mean. For continuous variables, one-way repeated measure analysis of variance (ANOVA) (followed by Tukey’s post hoc test) was used for multiple comparisons and two-tailed Student’s *t* test for two-group comparisons. For categorical variables, Pearson chi-square test was used. Differences between the experimental groups were considered statistically significant at *p* < 0.05.

## Results

All animals included in the present study were subjected to an 8-day period of habituation (Fig. 1a) before assessing their locomotor response to an incremental load test of forced wheel running [42, 43]. Typically, the locomotor performance is revealed by the amount of time spent running while the rotation speed of the wheel increases (Fig. 1b). Relative to the vehicle group, systemic (i.p.) administration of SCH23390 (0.1 and 0.2 mg/kg) or raclopride (0.5 and 1 mg/kg) alone reduced the time of running during the incremental test in a dose-dependent manner (Fig. 2a, b). Interestingly, the behavioral effects of SCH23390 and raclopride alone are comparable. Next, the administration of the lowest effective dose of SCH23390 (0.1 mg/kg) + raclopride (0.5 mg/kg) given together showed a marked locomotor disruption during the incremental test (Fig. 2c). Despite that the overall level of locomotor disruption observed with SCH23390 or raclopride alone is not statistically different from SCH23390 + raclopride (*p* = 0.053 vs SCH23390 or raclopride alone, one-way ANOVA), none of the animals from the latter group surpassed the 5-min running time (*p* < 0.0001 vs raclopride alone, *p* < 0.005 vs SCH23390 alone, chi-square test) (Fig. 2c). These data indicate that activation of D1 and D2 receptors are required to sustain the locomotor response to incremental loads of forced running. However, our data also revealed that systemic blockade of either D1 or D2 receptors independently is sufficient to disrupt the locomotor behavior in the open field, although a robust motor impairment emerges following systemic administration of SCH23390 + raclopride (Fig. 2d). Together, these results imply that both motor skills in the running wheel and locomotor activity in the open field are dopamine dependent.

We next asked whether the comparable locomotor disruption resulting from systemic administration of SCH23390 and raclopride when given alone shares a common anatomical target. Of particular interest is the striatum, one of the major targets of dopamine and well-known for its role in modulating motor behavior [19–23, 48, 49]. Thus, it is possible that the behavioral effects observed following systemic administration of dopamine antagonists occurred through the striatum. To test this hypothesis, we generated another cohort of rats where guide cannulas were placed into the dorsal striatum (Fig. 3a) and changes in the duration of forced running during the incremental test were assessed following striatal injections of SCH23390 or raclopride (Fig. 3b). Relative to vehicle infusions, striatal delivery of SCH23390 (10–100 μM) reduced the time spent during the incremental test (Fig. 3b). Interestingly, this was not the case following infusions of raclopride (20μM–5 mM) as revealed by the level of locomotor performance resembling that of the vehicle group (Fig. 3b). Of note, striatal injections of SCH23390 did not disrupt locomotor activity in the open field (Fig. 3c), which implies that striatal D1 receptor signaling is preferentially recruited during incremental loads of forced running. Furthermore, the lack of effect after intrastriatal administration of raclopride together with the observed systemic effect implies an extra-striatal D2 receptor signaling mechanism underlying the modulation of the behavioral response during the incremental test.

## Discussion

In the present study, we found that the level of locomotor response to incremental loads of forced running in adolescent rats is dopamine dependent and mechanistically linked to the activation of D1 and D2 receptors. Our data also show that striatal blockade of D1, but not D2 receptors, reduced the response during the incremental test. Together, these results imply a recruitment of striatal and extra-striatal D1 and D2 receptor signaling to sustain proper level of locomotor performance during forced running.

The dopaminergic system is known to play a key role in the development and maturation of neural circuits associated with cognitive and motor learning behaviors during adolescence [3, 15, 19–23, 41]. Accordingly, systemic administration of D1 and D2 receptor antagonists reduced the duration of forced running in a dose-dependent manner. Of note, both dopamine receptor antagonists elicited similar levels of locomotor disruptions when given alone, which is consistent with the idea of a synergistic D1-D2 action to maintain coordinated motor activity [50–53]. However, the behavioral impact of D1 and D2 receptor antagonists was not limited to reducing the response in the forced running wheel, as revealed by the attenuated locomotor activity in the open field and severe motor deficit when SCH23390 + raclopride were administered together. In fact, dopamine antagonists also impair motor coordination during voluntary wheel running [20, 32, 34, 54]. This suggests a common neural mechanism underlying the regulation of ambulatory locomotor activity and motor skills in the running wheel by dopamine. Future studies are warranted to determine whether the neural substrate modulating the motor response is age dependent, despite the fact that the results obtained in the open field from adolescent rats are similar to those induced by SCH23390 [33, 53, 55, 56] and raclopride [30, 31, 56, 57] in adults.

The striatum is the main input structure of the basal ganglia circuitry involved in motor control and one of the brain regions with the highest expression of dopamine receptors [15, 19–23, 44, 48, 49, 58]. Thus, the level of locomotor response to incremental loads of forced running is expected to be mitigated following striatal delivery of dopamine receptor antagonists. Our data revealed that only striatal D1 receptors are required to maintain proper levels of performance during forced running, whereas striatal D2 receptors are not. These findings are consistent with the prominent role of striatal D1 receptors over D2 receptors in the regulation of coordinated motor behavior [20, 59–64], including voluntary wheel running [20] and ambulatory activity [49, 65]. However, our results also suggest that there is an extra-striatal component modulated by D2 receptors that acts synergistically with the striatal D1 receptor signaling to sustain the behavioral response during incremental loads of forced running.

In our previous [42, 43] and current studies, the exposure to an 8-day period of habituation is critical to enable a higher locomotor performance in the forced running wheel [42, 43]. It is therefore conceivable that the dopaminergic system is also recruited during the habituation phase to enhance the level of motor coordination and response to incremental loads of running. While such a dopaminergic effect could be mediated by several cellular and synaptic mechanisms, activation of striatal D1 receptors can effectively potentiate corticostriatal transmission [62, 66–71] during habituation to promote motor coordination and motor skill learning [20, 59–61]. Remarkably, our data also point toward the contribution of extra-striatal D2 receptors [72–78] in facilitating the behavioral response, despite the fact that D1 and D2 receptors often exert opposing postsynaptic effects [24–26]. Certainly, D2 receptor deletion impairs locomotion, motor skill learning, and coordination [79–81], resembling the motor disruption elicited by chronic depletion of the nigrostriatal dopamine pathway and associated striatal deficit of D2 receptor signaling [79, 82–86]. Hence, it is possible that striatal and extra-striatal motor circuits are gradually recruited by D2 receptors during the habituation period [3, 72–78] to enable the potentiation of corticostriatal transmission and locomotor response by D1 receptors.

In addition to the dorsal striatum and dopamine, other motor-related neurotransmitter systems, such as serotonin [87], and extra-striatal structures are likely to interact in the regulation of exercise capacity. In this regard, the nucleus accumbens has been pointed as a critical component in modulating effort-related functions due to its role in decision-making and motivation [72, 88, 89]. Other candidate structures include the globus pallidus, since it has been highlighted to orchestrate dynamic aspects of basal ganglia regulation of motor coordination [90]. Although the integration of sensory and cognitive information by the striatum is required during movement initiation [58], increments in striatal activity are often preceded by convergent projections from multiple cortical areas, such as the motor cortex [76] and the prefrontal cortex. The latter cortical region is of particular interest for its importance in the neural maturation during adolescence [13, 14] as well as its role in working memory, planning, and executive functions [91].

Collectively, the results presented here indicate that coordinated modulation of striatal and extra-striatal neural circuits’ activity by dopamine could play a major role in adjusting the level of locomotor response during forced running. Whether similar neural mechanisms are recruited to enhance other forms of effort-related motor skill behaviors [3, 72, 89] remains to be determined.

## Data Availability

The dataset generated during the current study are available from the corresponding author on reasonable request.
